# Drug-Induced Lysosomal Impairment Is Associated with the Release of Extracellular Vesicles Carrying Autophagy Markers

**DOI:** 10.3390/ijms222312922

**Published:** 2021-11-29

**Authors:** Krizia Sagini, Sandra Buratta, Federica Delo, Roberto Maria Pellegrino, Stefano Giovagnoli, Lorena Urbanelli, Carla Emiliani

**Affiliations:** 1 Department of Chemistry, Biology and Biotechnology, University of Perugia, 06123 Perugia, Italy; krizia.sagini@studenti.unipg.it (K.S.); sandra.buratta@unipg.it (S.B.); federica.delo@studenti.unipg.it (F.D.); roberto.pellegrino@unipg.it (R.M.P.); carla.emiliani@unipg.it (C.E.); 2 Department of Surgery, Division of Cancer Biology and Therapeutics, Cedars-Sinai Medical Center, Los Angeles, CA 90048, USA; 3 Department of Pharmaceutical Sciences, University of Perugia, 06126 Perugia, Italy; stefano.giovagnoli@unipg.it; 4 CEMIN (Center of Excellence for Innovative Nanostructured Material), University of Perugia, 06123 Perugia, Italy

**Keywords:** phospholipidosis, lysosomal storage disorders, amiodarone, chloroquine, bafilomycin A1, extracellular vesicles, exosomes, microvesicles, secretory autophagy, TFEB

## Abstract

Amiodarone is a cationic amphiphilic drug used as an antiarrhythmic agent. It induces phospholipidosis, i.e., the accumulation of phospholipids within organelles of the endosomal–lysosomal system. Extracellular vesicles (EVs) are membrane-enclosed structures released by any type of cell and retrieved in every fluid of the body. EVs have been initially identified as a system to dispose cell waste, but they are also considered to be an additional manner to transmit intercellular signals. To understand the role of EVs in drug-induced phospholipidosis, we investigated EVs release in amiodarone-treated HEK-293 cells engineered to produce fluorescently labelled EVs. We observed that amiodarone induces the release of a higher number of EVs, mostly of a large/medium size. EVs released upon amiodarone treatment do not display significant morphological changes or altered size distribution, but they show a dose-dependent increase in autophagy associated markers, indicating a higher release of EVs with an autophagosome-like phenotype. Large/medium EVs also show a higher content of phospholipids. Drugs inducing lysosomal impairment such as chloroquine and bafilomycin A1 similarly prompt a higher release of EVs enriched in autophagy markers. This result suggests a mechanism associated with amiodarone-induced lysosomal impairment more than a connection with the accumulation of specific undigested substrates. Moreover, the implementation of the lysosomal function by overexpressing TFEB, a master gene regulator of lysosomal biogenesis, prevents the amiodarone-induced release of EVs, suggesting that this could be a feasible target to attenuate drug-induced abnormalities.

## 1. Introduction

Drug-induced phospholipidosis (DIPL) is an acquired lipid storage disorder due to the massive intracellular accumulation of phospholipids in multilamellar inclusion bodies within the lysosomal–endosomal system [1,2,3]. Most of DIPL inducers are cationic amphiphilic drugs (CAD) that, once inside the cells, are protonated in the acidic environment of lysosomes and are locked in, thus alkalinizing lysosomal milieu and thus hampering lysosomal enzyme activity. However, the molecular mechanisms leading to the prevalent accumulation of phospholipids are unclear. The accumulation of this lipid class could be caused either by the inhibition of phospholipid degradation or enhancement of their biosynthesis. Phospholipids are usually degraded into lysosomes and the direct drug-induced inhibition of lysosomal phospholipases, or the formation of nondegradable drug–phospholipid complexes have been reported as a possible cause of their accumulation [2,4]. Recently, DIPL has been associated with a release of lysosomal hydrolases, which can be monitored in the cell culture medium by enzymatic assay [5,6]. Lysosomal enzymes are synthesized in the endoplasmic reticulum (ER) and transported into the Golgi apparatus, where they are either sorted from the trans-Golgi network (TGN) to the endosomal/lysosomal system or released outside the cell, from where they may enter the endocytic pathway thanks to the presence of the mannose 6-phosphate (M6P) receptors on the plasma membrane. These receptors recapture lysosomal hydrolases, eventually delivering them to lysosomes [7]. The extracellular release of lysosomal hydrolases in DIPL could be due to their higher active secretion from the Golgi route, possibly associated with the blockade of enzymes transport to lysosomes via the M6P receptor [5] or to a lower recapture from outside the cell. They could also be the consequence, or the result, of the activation of TFEB, a pivotal regulator of lysosomal biogenesis and exocytosis, as a cell’s attempt to ameliorate lysosomal storage [8].

Extracellular vesicles (EVs) are membrane-delimited nanoparticles released outside the cell and characterized by heterogenous size and shape [9,10]. Vesicles originating from the plasma membrane are often referred to as microvesicles or ectosomes, while vesicles originating from the endosomal system are usually indicated as exosomes. Microvesicles and exosomes overlap in size distribution and density, with microvesicles being reported between 50 and 1000 nm and exosomes between 30 and 150 nm [11]. This issue explains why exosome and microvesicle separation still remains an unmet challenge [12]. For this reason, the definition of large/medium EVs (l/mEVs) for microvesicle-enriched vesicle populations and of small EVs (sEVs) for exosome-enriched vesicle populations has been proposed [11].

From a functional point of view, exosomes were initially considered as part of the cell scavenging machinery for waste removal during the reticulocytes to erythrocytes maturation process [13]. Later, an immunomodulatory and signaling role emerged [14]. Studies have reported that many cellular stressors prompt the secretion of EVs, including hypoxia [15] and senescence following γ-irradiation [16] or oncogene expression [17]. Pharmacological treatments have also been shown to modulate vesicle release, as EV secretion has been related to the transfer and propagation of drug resistance to chemotherapeutics, such as cisplatin [18] and docetaxel [19].

Amiodarone (AM) is a well-known phospholipidosis inducer [20] that prompts the release of lysosomal enzymes in the cell culture medium [5,6,7]. AM binds primarily to the hydrophobic moiety of phospholipids, thus impairing their degradation by inhibiting phospholipases [2]. Gene expression studies reported the reduction of lysosomal phospholipase level and lysosomal enzyme transport upon AM treatment, as well as the induction of phospholipid and cholesterol biosynthesis [21].

The effect of this CAD on EV release has been poorly investigated [22], and it is unclear whether EVs could contribute to alleviating the intracellular accumulation of phospholipids. A few studies have provided evidence that in pharmacological as well as genetical models of lysosomal storage disorders (LSDs), cells try to overcome lysosomal substrate accumulation by increasing EV secretion [23,24]. Lysosomal impairment could block the autophagosome–lysosome fusion, which is required for the autophagic degradation, prompting the release of EVs [25,26,27]. Exosome secretion ameliorates the lysosomal storage of cholesterol in Niemann–Pick Type C disease [28,29], and curcumin, an autophagy inducer, promotes exosome/microvesicle secretion that attenuates lysosomal cholesterol traffic impairment [30].

Based on such premises, the aim of this study was to assess the release of EVs upon AM treatment. For this purpose, we developed a HEK-293 cell model engineered to produce fluorescently labelled EVs by overexpressing the EV marker CD63 as a fusion protein with the fluorescent protein mCherry. CD63 is considered one of the best available markers for EVs, as it is highly enriched in sEVs, but it is also present in l/mEVs [12]. We observed that AM treatment induced an increased release of l/mEVs (obtained by centrifugation at 10,000× *g*, also termed 10K fraction). Moreover, both l/mEVs and sEVs released upon AM treatment contained a dose-dependent higher level of autophagy-associated markers and, in the case of l/EVs, a higher content of phospholipids, without changes in their morphology or size distribution. Drugs inducing lysosomal impairment such as chloroquine and bafilomycin A1 also prompted the release of a higher amount of EVs carrying autophagy markers. These findings suggested that a higher release of autophagy-associated structures upon AM treatment, indicating that a quantitative and qualitative alteration of EV secretion, is part of the cellular response to AM-induced lysosomal impairment. Implementing lysosomal system functionality by expression of its master regulator TFEB [8,31] prevented the higher release of l/mEVs upon AM treatment, indicating that this could be a valuable target to attenuate AM-induced alterations.

## 2. Results

### 2.1. Cells Overexpressing Fluorescent Proteins Release Fluorescent EVs

**Figure 1:**
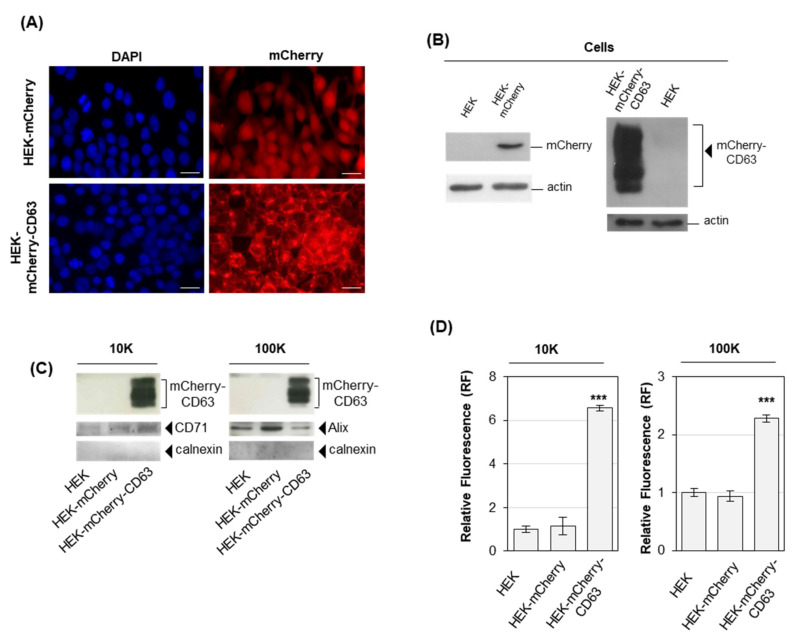
**HEK cells expressing mCherry release fluorescent EVs**. (**A**) Fluorescence microscopy. HEK cells were either transfected with mCherry or with mCherry-CD63 and selected with blasticidin-S. Cells were grown onto glass coverslips, fixed with 4% paraformaldehyde and stained for nuclei with DAPI. Magnification 20×. Scale bar 20 µm. (**B**) Immunoblotting analysis of mCherry in cells. Extracts were sized by SDS-PAGE and probed with an anti-mCherry antibody. As internal control, the membrane was also probed with an antiactin antibody. (**C**) Immunoblotting analysis of mCherry in EVs. Vesicles were obtained by centrifugation at 10,000× *g* (10K fraction) or 100,000× *g* (100K fraction). Extracts were sized by SDS-PAGE and probed with the indicated antibody. (**D**) Fluorescence analysis of EVs. 10K and 100K fraction pellets from the culture medium of 107 cells were resuspended in 100 µL PBS and assayed for fluorescence at 580 nm ex, 620 nm em. *** *p* < 0.001 with respect to untransfected HEK cells as control.

To set up a cell model allowing one to easily monitor EV release under treatment with phospholipidosis inducers, HEK-293 cells were transfected to constitutively express CD63 [11,12] as a fusion construct with the fluorescent protein mCherry [32]. Upon transfection, cells were selected with 10 µg/mL blasticidin-S until the stable expression of the fluorescent construct. HEK cells were also transfected with mCherry alone, as a control of subcellular localization. Analysis of HEK-mCherry-CD63 and HEK-mCherry by microscopy clearly showed a different fluorescence pattern, as mCherry fluorescence alone was diffused in all cell compartments, coherently with the absence of a localization signal, whereas mCherry-CD63 fluorescence was consistent with membrane localization [33] (Figure 1A). Immunoblotting on total cell extracts with an anti-mCherry antibody confirmed a high level of mCherry expression in both cell models, as compared to untransfected HEK cells, with a wide signal for HEK-mCherry-CD63 due to the presence of CD63 glycosylated forms (Figure 1B). EVs were purified by differential ultracentrifugation (dUC) (see Section 4), after 24 h incubation in a cell culture medium supplemented with exosome-depleted serum. In brief, the supernatant recovered after centrifugation at 2000× *g* was centrifuged again at 10,000× *g* (10K). Pelleted material at 10,000 g was washed, obtaining l/mEVs (10K fraction), whereas the supernatant was ultracentrifuged at 100,000× *g* (100K). Pelleted material at 100,000 g was washed, obtaining sEVs (100K fraction).

EV fractions were analyzed by immunoblotting (Figure 1C) and for fluorescence (Figure 1D). Immunoblotting on EVs showed that the mCherry-CD63 construct was highly expressed in 10K and 100K fractions from HEK-mCherry-CD63 cells (Figure 1C). HEK-mCherry-CD63 10K and 100K pellets showed a significant fluorescence increase compared to 10K and 100K pellets from HEK-mCherry and HEK cells (Figure 1D). These results indicated HEK-mCherry-CD63 as a cell model suitable to follow changes in the release of both l/mEVs and sEVs, upon their separation by dUC and assessment of 10K and 100K pellets fluorescence. A similar cell model obtained overexpressing CD63-EGFP in HEK cells failed to show significantly higher fluorescence in the 10K fraction EVs upon their purification (Supplementary Figure S1). Therefore, the HEK-mCherry-CD63 cell model was preferred.

**Figure 2:**
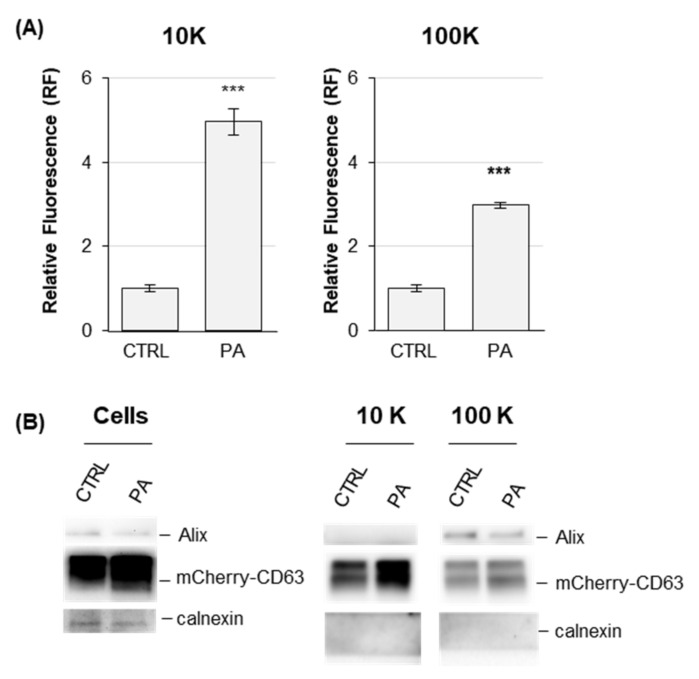
**Analysis of EVs released by in HEK-mCherry-CD63 upon palmitic acid (PA) treatment**. (**A**) Fluorescence analysis of EVs. L/mEVs (10K) and sEVs (100K) were prepared by dUC from condition media of HEK-mCherry-CD63 cells treated with 200 μM PA. Pellets were resuspended in 100 µL PBS and assayed for fluorescence at 580 nm ex, 620 nm em. Results represent the mean ± SE of three independent experiments performed in duplicate. *** *p* < 0.001 with respect to vehicle-treated cells as control. (**B**) Immunoblotting analysis of cells and EVs treated with PA. For cells, extracts were sized by SDS-PAGE and probed with an anti-mCherry antibody, stripped and probed again with anti-Alix and anticalnexin antibodies. As an internal control, the membrane was also probed with an antiactin antibody. For EVs, extracts were sized by SDS-PAGE and probed with an anti-mCherry antibody, stripped, and probed with the indicated antibodies.

To further confirm HEK-mCherry-CD63 cells as a suitable cell model to monitor changes in the release of EVs, cells were incubated with palmitic acid (PA), which is known to prompt the release of EVs [34,35]. The treatment with 200 µM PA, although nontoxic as assessed by MTT and LDH assays (data not shown), increased the fluorescence of 10K and 100K pellets up to 5 times and 3 times, respectively, as compared to EVs purified from vehicle-treated HEK-mCherry-CD63 as control (Figure 2). The presence of negative EV marker such as calnexin was not detected upon PA treatment (Figure 2B). This result indicated that the increased release of both l/mEVs and sEVs releases induced by PA treatment could be monitored in HEK-mCherry-CD63 cells by separating 10K and 100K fractions via dUC and then measuring their fluorescence, thus validating our model.

### 2.2. Amiodarone Affects the Release of Large/Medium and Small EVs in a Dose-Dependent Manner

**Figure 3:**
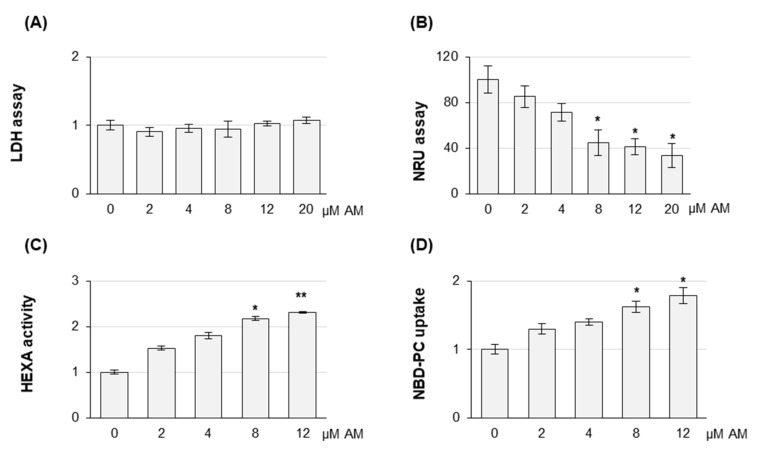
**Effect of AM treatment on HEK-mCherry-CD63 cells**. Cells were treated for 24 h with AM at the indicated concentrations. In all assays, except for NR uptake (NRU) in (**B**), the results are reported as fold induction with respect to vehicle-treated cells as control (set 1). For NR uptake (NRU), results are reported as percentage with respect to vehicle-treated cells as control (set 100). (**A**,**B**), Viability of HEK-mCherry-CD63 cells determined by LDH (**A**) and NRU (**B**) assays. Data are the mean ± SE of three independent experiments, each performed in triplicate (* *p* < 0.05, with respect to drug-treated cells vs. vehicle controls). (**C**) Lysosomal β-hexosaminidase A (HexA) isoenzyme activity in culture medium. Data represent the mean ± SE of at least three independent experiments, each one in duplicate (* *p* < 0.05, ** *p* < 0.01, drug-treated cells vs. vehicle as control). (**D**) NBD-PC uptake in HEK-mCherry-CD63. NBD-PC fluorescence was measured at 485 nm Ex/538 nm Em, and then nuclei were stained with Hoechst 33,342 and fluorescence was measured at 355 nm Ex/460 nm Em. Normalized values were calculated as the ratio between the NBD-PC and the Hoechst 33,342 values. Results represent the mean ± SE of three independent experiments performed in triplicate (* *p* < 0.05, drug-treated cells vs. vehicle as control).

Before assessing the effect of AM on EV release, AM-induced toxicity was investigated on HEK-mCherry-CD63. No increase in LDH activity could be detected in HEK-mCherry-CD63 cell culture media up to 20 µM AM treatment (Figure 3A), whereas the NRU assay, which is correlated with lysosomal functionality, clearly showed a significant dye uptake decrease at 8 µM AM concentration (Figure 3B). Similar results were obtained for HEK-mCherry and HEK cells as control, indicating that AM displayed similar effects on the 3 cell lines and that cell viability was not significantly affected by mCherry-CD63 overexpression (data not shown). We previously demonstrated that AM-induced phospholipidosis is associated with the extracellular release of the lysosomal enzyme β-hexosaminidase (HexA) and that phospholipid accumulation can be measured by detecting the cell accumulation of the synthetic fluorescent lipid NDB-PC [6,8]. When these assays were carried out on HEK293 cells, a significant increase in HexA activity and the accumulation of the fluorescent phospholipid NDB-PC were observed starting at 8 µM AM (Figure 3C,D). The absence of significant extracellular LDH activity at this concentration indicated that the increase in HexA enzyme activity was unrelated to membrane damage and intracellular content release. Moreover, the reduced NR uptake into lysosomes already at 8 µM indicated that at this concentration AM induced an impairment of lysosomal function, which is associated with a significant release of HexA and the accumulation of the NDB-PC fluorescent lipid. Similar results were obtained when AM was used to treat HEK-mCherry and untransfected HEK (data not shown).

**Figure 4:**
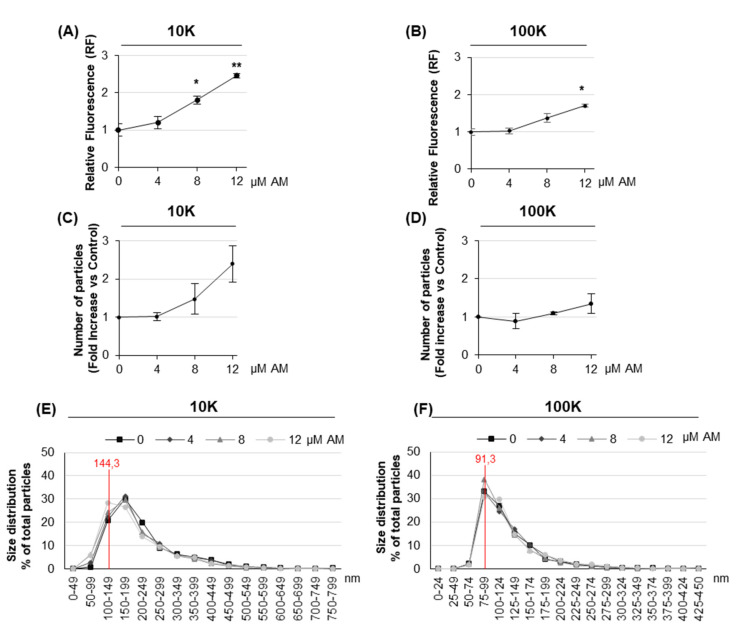
**Analysis of EVs released by HEK-mCherry-CD63 upon AM treatment**. (**A**,**B**) Fluorescence analysis of EVs. EVs were obtained from cell culture media by dUC at 10,000 g (10K fraction) and 100,000 g (100K fraction). Pellets were resuspended in 100 µL PBS and assayed for fluorescence at 580 nm ex/620 nm em. Values are the mean ± S.E. of at least three experiments, each one in duplicate. * *p* < 0.05, ** *p* < 0.01 with respect to vehicle-treated cells as control (set 1). (**C**,**D**) Quantification of EVs. The amount of EVs in 10K and 100K fractions was measured by NTA. EV pellets were resuspended in 0.02 μm filtered PBS to obtain a concentration within the recommended range (2 × 10^8^–1 × 10^9^ particles per ml) and then loaded into a NS500 instrument. Data were calculated as number of particles/cell and reported as fold increase with respect to vehicle-treated cells as control (set 1). Values are the mean ± S.E. of at least three experiments, * *p* < 0.05, with respect to vehicle-treated cells. (**E**,**F**) Particle size distribution of 10K and 100K EVs, reported as percentage particle, i.e., the percentage of particles of the indicated diameter with respect to the total number of analyzed particles. Values are the mean of at least three experiments. The red line indicates the mode value of 10K and 100K EVs.

To investigate the effect of AM on EV release, HEK-mCherry-CD63 were treated with increasing concentration of the drug. Both l/m and sEVs were isolated by dUC as described above, resuspended in PBS and their associated fluorescence measured. Results showed that AM induced a dose-dependent fluorescence increase in the 10K fraction, already significant at 8 µM (Figure 4A), while it increased the 100K fluorescence only at 12 µM (the higher concentration being tested) (Figure 4B). In parallel, we counted the number of released EVs by NTA (Figure 4C,D). For the 10K fraction, the higher level of EVs fluorescence detected upon AM treatment was mirrored by a larger number of secreted EVs. Therefore, the endosomal–lysosomal perturbation induced by AM clearly affects the release of l/mEVs in terms of vesicle fluorescence and quantity. Moreover, it has a smaller but significant effect also in sEV release, as detected by fluorescence measurement. NTA analysis also allowed the monitoring of the size distribution of both the 10K and 100K EVs. Results showed that the 10K fraction contained vesicles having a modal value of 144.3 nm, whereas the 100K fraction showed a modal value of 91.3 nm. In addition, it provided evidence that there is no difference in terms of size distribution between vehicle-treated cells and cells treated with 4, 8, and 12 µM AM, both in the case of the 10K (Figure 4E) and 100K fraction (Figure 4F).

**Figure 5:**
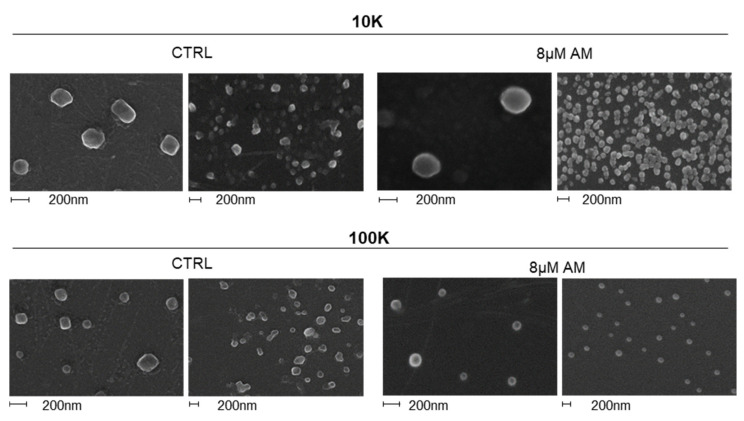
**Scanning electron micrographs of EVs**. EVs were isolated by dUC, fixed with 2.5% glutaraldehyde in PBS, sedimented onto glass coverslips, allowed to dry at room temperature. Images were obtained using a field emission gun electron scanning microscope after Cr metallization.

We also assessed EV morphology by SEM analysis (Figure 5). Results confirmed no major changes in EV size and morphology for both fractions between AM treated and controls, although in terms of morphology vesicles from AM-treated samples, they appeared more regularly rounded shaped.

### 2.3. Amiodarone Modifies the Content of Large/Medium EVs

**Figure 6:**
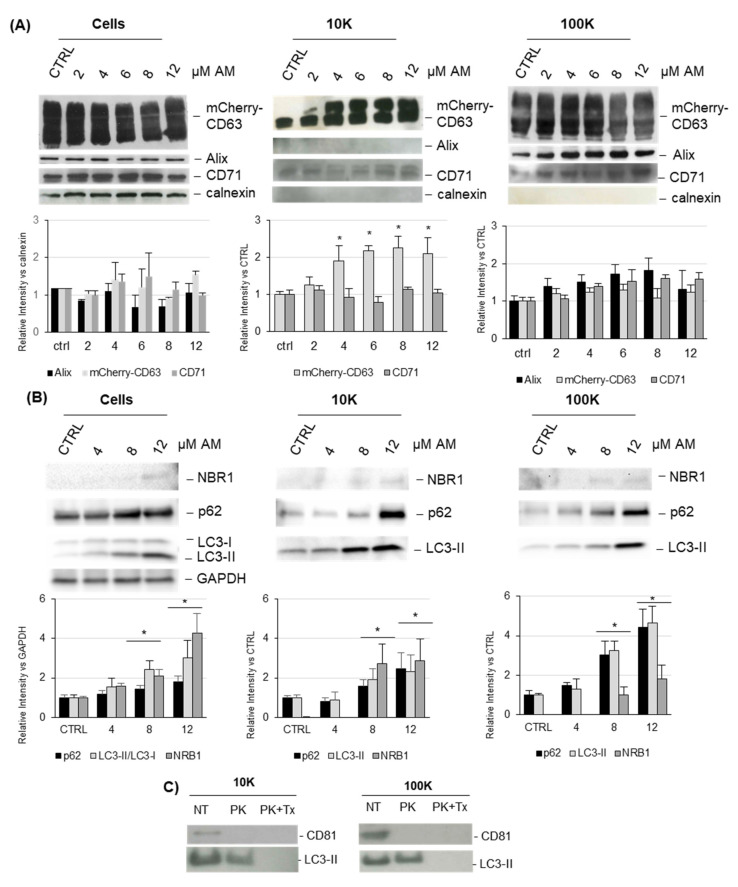
**Characterization of EVs released from HEK-mCherry-CD63 upon AM treatment**. Cell extracts (30 μg) and EV preparations (3 μg) were separated by SDS-PAGE, electrotransferred, and then probed with the indicated markers (Panel (**A**) and with autophagy markers (Panel (**B**)). The densitometric analysis of results is reported below. In cell extracts, bars represent the ratio between the intensity of the band signal and calnexin (Panel (**A**), **left**) or GAPDH (Panel (**B**), **left**). In EVs, bars represent the ratio between the intensity of the band signal of AM-treated sample and vehicle-treated sample as control (set 1). When the signal was not detected in the vehicle-treated sample, the lowest intensity band detected was set at 1. Bars are reported as relative intensity. Results represent the mean ± SD of three independent experiments (* *p* < 0.05, drug-treated sample vs. vehicle as control). (**C**) Proteinase K protection assay of EVs isolated from 8 µM AM-treated cells. Vesicles of the 10K and 100K fraction were isolated from cell medium and divided into 3 aliquots: NT (not treated, i.e., not incubated with Proteinase K), PK (incubated with 50 µg/mL Proteinase K), and PK + Tx (incubated with 50 µg/mL Proteinase K and 0.5% Triton X-100). After 1 h incubation at 37 °C, Proteinase K was inactivated with 5 mM PMSF, and then samples were loaded on SDS-PAGE and blotted with the indicated antibodies.

Changes in protein marker composition were investigated in vesicles from AM-treated samples. Cell extracts and vesicles were tested for the presence of mCherry-CD63 and for the EV markers Alix and CD71 (Figure 6A). As expected, a high mCherry-CD63 level was detected in cells extracts as well as in the 10K and 100K fractions. Alix is a protein associated with the ESCRT complex and is involved in the biogenesis of exosomes and less clearly in the budding of microvesicles; therefore, it was expected to be detected mostly in the 100K fraction (enriched in exosomes) [36]. CD71, also known as p90 or transferrin receptor, is a plasma membrane protein recycled through the endosomal system, and it was predictable to be more abundant in the 10K fraction (enriched in microvesicles) [37]. Results showed that Alix was clearly detected in the 100K fraction but was not in the 10K fraction, whereas CD71 was clearly present in the 10K fraction but was also detected in the 100K fraction. It was previously demonstrated that AM induced the activation of autophagy and the accumulation of LC3 in treated cells, together with an increased conversion of LC3-I in LC3-II [8]. Based on such evidence, cells and EVs (10K and 100K fractions) were tested for the presence of autophagy markers (Figure 6B). Interestingly, we observed that both 10K and 100K fractions contained the lipidated LC3-II and displayed a clear dose-dependent increase in LC3 signal. This result indicated that upon AM treatment cells release EVs containing a higher level of LC3-II, normally associated with autophagosomal membranes. The well-known autophagy markers p62 and NBR1 were also increased in a dose-dependent manner in both 10K and 100K fractions upon AM treatment, which further supports the evidence the AM-induced the secretion of EVs with an autophagosome-like phenotype.

To assess the topological localization of LC3-II and rule out the possibility of a its co-precipitation outside EVs, we carried out the Proteinase K (PK) protection assay on both 10K and 100K EVs isolated from 8µM AM-treated cells (Figure 6C). Following their isolation, vesicles were divided into three aliquots: the first one was used as control; the second was treated with PK alone; and the third with PK together with the detergent Triton X-100 (PK + Tx) to permeabilized EV membrane. The three aliquots were then blotted, and results showed that the control protein CD81 tetraspanin, which is an integral membrane protein used as digestion control, was degraded by PK alone (and PK + Tx, as expected), whereas the autophagy marker LC3-II was only digested by PK + Tx alone, thus indicating that it is localized inside EVs, as it is protected from proteolytic digestion unless the membrane is solubilized.

**Figure 7:**
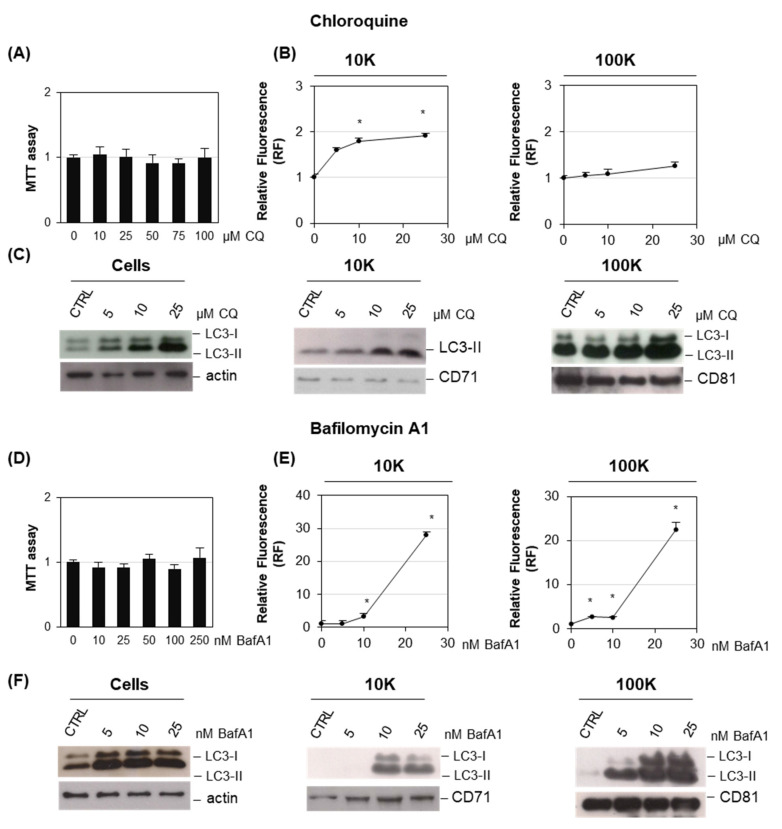
**Analysis of EVs release by HEK-mCherry-CD63 upon chloroquine (CQ) and bafilomycin A1 (BafA1) treatment**. (**A**,**D**) Viability of HEK-mCherry-CD63 cells determined by MTT assay. Data are the mean ± SE of three independent experiments, each performed in triplicate. (**B**,**E**) Fluorescence analysis of EVs. EVs were obtained by centrifugation at 10,000× *g* (10K fraction) and 100,000× *g* (100K fraction). Pellets were resuspended in 100 µL PBS, transferred on a 96-well microplate and assayed for fluorescence at 580 nm ex/620 nm em. Values are the mean ± S.E. of at least three experiments, each one in duplicate. * *p* < 0.05,with respect to vehicle-treated cells as control (set 1). (**C**,**F**) Characterization of EVs associated markers. Cell extracts (30 μg) and EV preparations (3 μg) were separated by SDS-PAGE, electrotransferred, and probed with the indicated antibodies.

To understand whether the release of EVs enriched in autophagy markers was related to AM-induced phospholipid accumulation or to a general lysosomal impairment, we tested the effect of two well-known lysosome perturbation agents: chloroquine (CQ) and bafilomycin A1 (BafA1) on the release of EVs (Figure 7) [38,39]. Our results clearly showed that at nontoxic concentrations (as tested by MTT assay), CQ and BafA1 both induced a dose-dependent increased release of l/mEVs, as measured by EV fluorescence. The increase was remarkably higher for BafA1 (about 20-fold) than for CQ (two-fold, such as AM). BafA1 also induced a significant increased release of sEVs (about 20-fold). Moreover, both drugs induced a dose-dependent increase in the autophagy-associated marker in l/mEVs and sEVs, as shown by immunoblotting. These results clearly indicated that the increased release of EVs carrying autophagy markers was a feature of lysosomal impairment.

**Figure 8:**
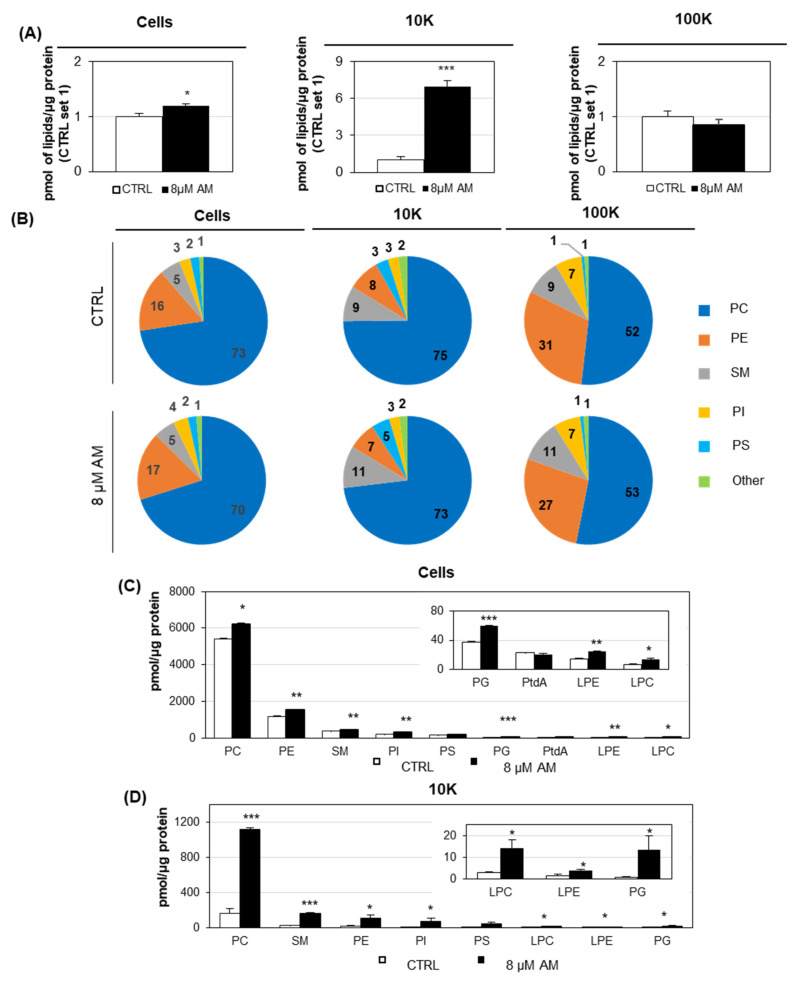
**Phospholipid composition of AM-treated vs. control samples**. Cells were incubated with 8µM AM for 24 h, and then 10K and 100K EVs were isolated from cell culture medium by dUC. Phospholipid content of AM-treated and control cells, and their released 10K and 100K EVs, were analyzed by LC/MS. (**A**) Relative amount of phospholipid normalized for protein content for cells (**left**), 10K EVs (**middle**), and 100K EVs (**right**). Data are reported as mean ± S.E. of three independent experiments, each in duplicate (* *p* < 0.05, ** *p* < 0.01, *** *p* < 0.001, AM-treated vs. vehicle-treated cells). (**B**) Phospholipid subclasses composition of AM-treated vs. control cells and their released EVs. The amount of each phospholipid subclass is expressed as the percentage of the sum of all identified lipids. The category indicated as “Other” includes lysophosphatidylcholine (LPC), lysophosphatidylethanolamine (LPE), phosphatidic acid (PtdA) and phosphatidylglycerol (PG). (**C**,**D**) Comparison of phospholipid subclasses composition of AM-treated vs. control cells and their released 10K EVs. Data are reported as mean ± S.E. of three independent experiments, each in duplicate (* *p* < 0.05, ** *p* < 0.01, *** *p* < 0.001, AM-treated vs. vehicle-treated cells). The inserted panels expand the vertical axis to allow the comparison of low abundance phospholipid subclasses.

The increased release of EVs, mostly of large/medium size, upon AM treatment, suggested that phospholipids themselves could be secreted within EV to dispose of excess lysosomal lipids. Changes in phospholipid content were therefore analyzed in cells treated with 8 µM AM and control cells, as well as in 10K and 100K fraction EVs. Data comparison highlighted numerous differences, but the most interesting variation was the total level of phospholipids normalized for protein content, which was seven-fold higher in 10K fraction released from AM-treated cells, with respect to the same fraction from untreated cells as control (Figure 8A). No significant difference was observed in the quantity of phospholipids, normalized for protein content of 100K fraction isolated from AM-treated and control cells, whereas for cells, the total amount of lipids was slightly increased in AM-treated samples. The three types of samples showed a specific composition, independently from the AM treatment (Figure 8B). As for example, sphingomyelin (SM) was the second most abundant lipid subclass in 10K EVs (independently from AM-treatment) but not in cells. Analysis of phospholipid subclasses revealed that all of them were significantly increased in l/mEVs released from AM-treated samples, except for phosphatidylserine (PS) (Figure 8D). As for cells, all phospholipid subclasses were present at a higher level in AM-treated cells, except for PS and phosphatidic acid (PtdA) (Figure 8C).

**Figure 9:**
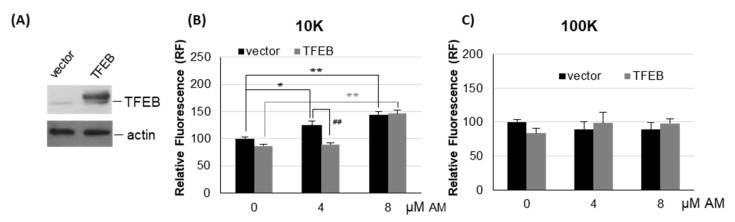
**Effect of AM on EVs release in HEK-mCherry-CD63 transfected with TFEB**. (**A**) Immunoblotting of cells transfected with TFEB. Extracts from cells transfected with TFEB or empty vector as control (vector) were incubated with an anti-TFEB antibody. As internal control, an anti-β-actin antibody was used. (**B**,**C**) HEK-mCherry-CD63 cells transfected with TFEB or empty vector as control (vector) were incubated for 24 h with 4 and 8 µM AM. EVs were obtained by culture medium centrifugation at 10,000× *g* (10K) and 100,000× *g* (100K). Pellets were resuspended in 100 µL PBS and assayed for fluorescence at 580 nm/620 nm (ex/em). Results represent the mean ± SE of three independent experiments performed in duplicate and are the reported percentage of empty vector transfected/vehicle-treated cells as control (set 100). * *p* < 0.05, ** *p* < 0.01, vector transfected drug-treated cells vs. vehicle, as control; ** *p* < 0.01, TFEB transfected drug-treated cells vs. vehicle as control; ^##^ *p* < 0.01, TFEB vs. empty vector transfected cells as control.

Previous studies demonstrated that the overexpression of the lysosomal master regulator TFEB was able to delay AM-induced lipid accumulation in hepatocytes [8]. We therefore analyzed whether TFEB overexpression also affected the release of EVs during AM treatment. We transfected cells with TFEB or with empty vector alone as control, and then we measured EVs associated fluorescence upon 4 µM and 8 µM AM treatment (Figure 9). Results showed that overexpression of TFEB did not significantly affect the secretion of l/mEVs and sEVs in untreated cells. Moreover, there were no significant differences in the release of sEVs (100K) between TFEB and empty vector transfected cells upon AM treatment. However, upon 4 µM AM treatment, cells overexpressing TFEB released significantly less l/mEVs (10K) as compared to control cells, whereas at a higher dose (8 µM) the behavior was identical. These findings indicate that overexpressing a lysosomal biogenesis master regulator such as TFEB has the potential to delay and/or prevent the AM-induced release of EVs.

## 3. Discussion

Phospholipidosis is a major concern for drug development, as intracellular accumulation of undigested phospholipids in lamellar bodies is a major source of side effects for different tissues [1]. Amiodarone is considered to be one of the most effective antiarrhythmic drugs, and it accumulates in tissues during chronic dosing, namely in liver [20,40]. Over the last decade, EVs have been identified as an additional means of intercellular communication but also as a manner to discard unwanted or unnecessary material, as for example during cell differentiation. Here, we investigated whether they could play a role in the cell response to CAD-induced phospholipid accumulation, possibly contributing to discard deposits and alleviate this phenotype. First, we developed a cell model suitable for following the release of EVs through monitoring the EV fluorescence, which could be useful to screen EV release upon treatment with different drugs inducing phospholipidosis and/or lysosomal impairment. Our cell model was based on the overexpression in HEK cells of the mCherry-CD63 construct, which fuses the EV marker CD63 with the fluorescent protein mCherry and allows one to follow the release of both large/medium (10K fraction) and small (100K fraction) fluorescent EVs upon their separation from the cell culture medium by dUC. AM treatment increased the secretion of l/mEVs and, to a lower extent, the release of sEVs, as measured by EV-associated fluorescence. In parallel, the same increase was confirmed by NTA counting for the 10K fraction but not for the 100K fraction. This result validated our model and reinforced the evidence that an increase in EV release is part of the cell response to phospholipid accumulation and lysosomal impairment induced by AM treatment. In addition, it agrees with a recent study reporting that AM-induced endosomal dysfunction increases EV secretion in human urine [41]. Phospholipid accumulation, HexA activity increase in cell culture medium and l/mEVs higher secretion were all significant at 8 µM A. This finding indicates that increased l/mEVs release is an early detectable event, whose sensitivity can be compared with other commonly used biomarkers, such as NBD-PC phospholipid accumulation and HexA activity in cell culture medium. Moreover, the measure of l/mEVs-associated fluorescence reflects l/mEVs counting by NTA, but it is certainly more adaptable to a high-throughput platform.

**Figure 10:**
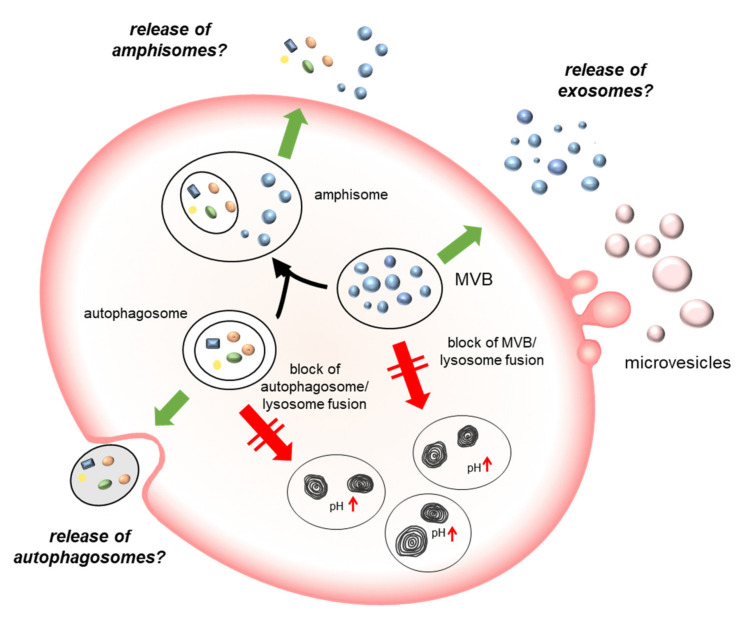
**Schematic representation showing the mechanisms of DIPL and EVs release**. The lysosomal dysfunction induced by the presence of undigested substrates is associated with the release of EVs enriched in autophagy markers. In the diagram, it is hypothesized that the reason may be an increased release of autophagososomes, because of the block of autophagosome/lysosome fusion, or of amphisomes, due to the block of amphisome/lysosome fusion. The release of exosomes may be induced as response to the block of MVB fusion with lysosomes and microvesicle secretion enhanced to maintain plasma membrane homeostasis after the increment of fusion events with organelles of the autophagy/lysosomal system. MVB, multivesicular body.

In addition to a quantitative evaluation, we also examined EVs released upon AM treatment from a qualitative point of view. NTA analysis showed that no alteration in terms of size distribution could be observed upon AM treatment, neither for l/mEVs nor for sEVs. SEM analysis allowed us to further check EVs from a morphological point of view, confirming no significant difference between EVs released by AM-treated and control cells. Previous studies reported that lysosomal status has an impact on EV release [25], and drugs activating autophagy such as curcumin [30] could alleviate intracellular accumulation of undigested substrates, prompting the release of EVs. Moreover, AM treatment was previously shown to induce an autophagic response in hepatocytes although not enough to prevent phospholipid accumulation [8]. The analysis of the autophagy markers LC3, p62 and NBR1 in AM-treated cells and their released EVs clearly revealed a dose-dependent increase in these markers not only in cells, as expected, but also in the 10K and 100K vesicles. Of relevance, the enrichment in autophagomal markers induced by AM treatment was found in both the 10K and 100K fractions, but other EV markers such as Alix and CD71 did not significantly change following AM treatment. This feature could be due to the possibility that AM treatment mostly affects the autophagic machinery dependent release of l/m EVs (Figure 10), or EV shape and density, allowing sedimentation at lower speed, as also suggested by the higher lipid content of l/mEVs. In fact, the lipidomic analysis of cells and both EV fractions, from AM-treated and control samples, clearly showed that 10K EVs from AM-treated samples were characterized by a higher content of phospholipids, indicating that their accumulation could be at least partially alleviated by releasing them extracellularly.

Studies have reported that cells use the autophagic structures, such as autophagosomes, not only to deliver cargo to be degraded into lysosomes, but also to dispose waste extracellularly, in an autophagic machinery dependent manner defined as “secretory” autophagy [28,42,43]. In addition, autophagosome can also fuse with MVBs to produce organelles known as amphisomes. These organelles have the features of both late endosomes and autophagosomes; in turn, they can either fuse with lysosomes for degradation or be released extracellularly [44]. Our results indicate that the AM-induced phospholipidosis is associated with the dose-dependent accumulation of the lipidated form of LC3, i.e., LC3-II in cells and EVs. Analysis of the topological localization of LC3 demonstrated that the protein is localized within EVs, as it is protected from Proteinase K digestion unless a detergent solubilizing membrane is added. The accumulation of LC3-II could be due to an impairment of lysosomal degradative function which prevents autophagosome/lysosome fusion and LC3-II recycling. AM, as well as other CADs, is known to induce the impairment of lysosome degradative function, mostly because of lysosomal alkalinization [40]. To unravel whether the higher release of EVs carrying an autophagy marker was specifically associated with the AM-induced accumulation of phospholipids or was a general response to lysosomal impairment, we investigated the effect of other drugs inducing lysosomal impairment without the accumulation of undigested substrates at the concentration tested, such as chloroquine and BafA1. Chloroquine has been previously associated with phospholipidosis at high doses, but at low concentration, it is commonly used to block autophagosome-lysosome fusion [38], whereas BafA1 is known to disrupt autophagic flux by independently inhibiting V-ATPase-dependent acidification and Ca-P60A/SERCA-dependent autophagosome-lysosome fusion [39]. Both drugs prompted a higher release of EVs of the 10K fraction, and notably for BafA1 this increase was quantitatively remarkable and clearly detected also for the 100K fraction, whereas for CQ, the effect was quantitatively comparable to that of amiodarone. However, both drugs induced the release of 10K and 100K fraction EVs characterized by a dose-dependent increase in the autophagy marker LC3. These results clearly indicated that these features were due to AM-induced lysosomal impairment and not to the accumulation of specific undigested substrates. They further suggest that BafA1 is specifically able to stimulate the release of EVs specific mechanisms that need further investigation to be elucidated.

Once established that the perturbation on EV release is a feature of AM-induced phospholipidosis and lysosomal impairment, we examined whether the implementation of lysosomal function by overexpression of the lysosomal master regulator TFEB could halt the increased EV release associated with AM treatment This gene induces lysosomal biogenesis and autophagy, preventing the intracellular accumulation of phospholipids as previously demonstrated [8]. Results showed that TFEB overexpression has a significant effect of decreasing the release of l/mEVs induced by AM treatment, but only at low AM concentration, whereas at a higher concentration, the behavior was not significantly different. Of note, TFEB overexpression did not affect either l/mEV or sEV release in untreated cells. This result indicates that when the lysosomal degradative capacity is high, cells do not need to increase EV release to alleviate stress associated with undigested substrate accumulation. Although other more specific alteration in EVs content in terms of lipids, proteins, and nucleic acids upon TFEB expression cannot be excluded, results reinforce the evidence that increasing the autophagic capacity of the cell favors autophagosome/lysosome fusion instead of secretion, decreasing the recovery of EVs [8].

The finding that lysosomal perturbing agents, such as AM, prompts mostly the release of l/mEVs may have pathological implications. Indeed, phospholipidosis has been defined as an “acquired” lysosomal storage disorders (LSDs), but it is reasonable to assume that in other LSDs of genetic origin [2] or in pathologies characterized by accumulation of undigested substrates into lysosomes, such as neurodegenerative diseases, EVs altered release could contribute to copathological features, such as abnormal development and inflammation [45]. As a matter of fact, in the case of Niemann–Pick disease, EV secretion has been reported to ameliorate lysosomal storage of cholesterol, although the impact of these EVs on the neighbor tissue is unknown [29]. In summary, our findings indicate that a higher level of EV release is a feature associated with AM-induced phospholipidosis and lysosomal impairment in vitro, and the biochemical content of these vesicles indicates a crosstalk between secretory autophagy and EV secretion. However, further studies are needed to correctly identify the exact cellular origin of these LC3-II positive vesicles released extracellularly upon phospholipidosis induced by AM and their role in the side effects reported for the drug.

## 4. Materials and Methods

### 4.1. Constructs

Full-length human CD63 was subcloned from CD63-pEGFP C2 (a gift from Paul Luzio, Addgene plasmid #62964) to pcDNA™6/myc-His A vector (Invitrogen, Waltham, MA, USA) to produce CD63 without tag using KpnI–BamHI enzymes. Then, mCherry sequence was obtained by PCR from the plasmid mCherry-hALIX (a gift from James Hurley, Addgene plasmid # 21504 [46] using 5′-GGG TAC CAT GGT GAG CAA GGG CGA GGA G (forward) and 5′-GGC ATG GAC GAG CTG TAC AAG TGG TAC CCC (reverse) primers and cloned into the pcDNA™6/myc-His A vector (Invitrogen) in frame with CD63 to produce mCherry-CD63 using KpnI enzyme. The previously mentioned mCherry sequence was also cloned into pcDNA™6/myc-His A vector to produce mCherry protein alone.

### 4.2. Cell Culture and Transfection

HEK-293 cells (ATCC, Manassas, VA, USA) were cultured in DMEM supplemented with 10% (*v*/*v*) heat-inactivated fetal bovine serum, 2 mM L-glutamine, 100 units/mL penicillin, 100 mg/mL streptomycin in a humidified incubator under 5% CO_2_ at 37 °C. Cell viability was estimated by examining their ability to exclude trypan blue 0.1% (*v*/*v*) in 0.9% (*v*/*v*) NaCl. To obtain stable cell lines expressing the fluorescent proteins, cells were seeded in six-well plates at 70% confluence and transfected with 1 μg/well of plasmid DNA using 3 μL/well of Lipofectamine LTX, according to manufacturer’s instructions. Transfected cells (HEK-mCherry, HEK-mCherry-CD63) were diluted and selected with 10 µg/mL blasticidin-S (Sigma-Aldrich, St. Louis, MO, USA).

### 4.3. Extracellular Vesicle Purification

For EVs isolation from condition media, cells were cultivated for 24 h in medium supplemented with exosome-depleted serum (System Biosciences, Palo Alto, CA, USA). For experimental purposes, cells were treated with different concentrations of amiodarone (AM, Sigma-Aldrich), palmitic acid (PA, Sigma-Aldrich), chloroquine (CQ, Cell Signaling Technology, Danvers, MA, USA), bafilomycin A1 (BafA1, Cell Signaling Technology) or vehicle (0.05% DMSO, CTRL) for 24 h in the same conditions. Medium was collected and underwent serial centrifugation steps to remove cells and cell debris (300× *g*, 10 min and 2000× *g*, 10 min, respectively). L/m EVs were recovered by centrifugation at 10,000× *g* for 30 min (10K fraction), and the pellet was washed with PBS and then centrifuged again at 10,000× *g*, resuspended in PBS and stored at −80 °C. For sEVs recovery, the supernatant obtained upon 10,000 g centrifugation was ultracentrifuged at 100,000× *g* for 70 min (100K fraction), the resulting pellet washed with PBS, resuspended in PBS and stored at −80 °C. All PBS was 0.22 µm filtered. Protein content was determined by the Bradford method, using bovine serum albumin as standard.

#### 4.3.1. Extracellular Vesicle Fluorescence Measurement

For fluorescence measurement, EVs were recovered as described in Section 4.3, then resuspended in the same amount of PBS and transferred into a 96-well black plate (Greiner, Frickenhausen, Germany). Fluorescence was measured on an Infinite^®^ 200 PRO microplate reader (TECAN, Männedorf, Switzerland), at excitation and emission wavelengths of 580 and 620 nm, respectively.

#### 4.3.2. Extracellular Vesicle Scanning Electron Microscopy

EVs were fixed in 2.5% glutaraldehyde for 15 min at RT, washed twice with large volume of water using concentration devices (Vivaspin, 300,000 Da cut-off, SARTORIUS, Goettingen, Germany). EVs were sedimented on glass coverslips and then dried at RT. Images were obtained using a field emission gun electron scanning microscope (LEO 1525 Zeiss; Thornwood, NY, USA). Cr metallization was carried out with a high-resolution sputter Q150T ES apparatus (24 s sputter at a current of 240 mA, Quorum, Lewes, UK). The thickness of chromium was ~10 nm

#### 4.3.3. Extracellular Vesicle Nanoparticle Tracking Analysis

EV pellets were resuspended in PBS (filtered through a 0.02 μm filter) at a concentration within the recommended range for particle count (2 × 10^8^–1 × 10^9^ particles per ml), then vortexed for 1 min. Samples were loaded into a NS500 instrument (Malvern Panalytic, Malvern, UK). Five videos, each of 60 s, were acquired for every sample, and the analysis was carried out by NTA 2.3 software. Releasing cells were detached and counted using Countess™ II Automated Cell Counter (Applied Biosystems™, Waltham, MA, USA). The total number of particles was normalized to the total number of releasing cells and expressed as fold with respect to vehicle-treated cells (0.05% DMSO). Particle size distribution was reported as percentage particle, i.e., the percentage of particles of the indicated diameter with respect to the total number of analyzed particles.

#### 4.3.4. Extracellular Vesicles Proteinase K Protection Assay

EVs fractions were isolated by dUC as described above and then resuspended in approximately 90 µL of PBS. The sample was divided into 3 identical aliquots: not treated (NT), treated with 50 µg/mL Proteinase K alone, treated with 50 µg/mL Proteinase K and 0.5% Triton X-100. The 3 aliquots were incubated for 1 h at 37 °C, and then, Proteinase K activity was inhibited by adding 5 mM of PMSF. Aliquots were mixed with 5× loading buffer containing 125 mM DTT and stored at −20 °C until gel loading. Immunoblotting was carried out as described below.

### 4.4. LDH Assay

The effect of AM treatment on cell viability was assessed by lactate dehydrogenase (LDH) leakage. LDH activity was determined in extracellular medium with CytoTox 96^®^ Non-Radioactive Cytotoxicity Assay kit (Promega, Madison, WI, USA), according to manufacturer’s instructions. In brief, the same cell medium preparations used for fluorescence measurements were transferred into a clear 96-well plate and CytoTox 96^®^ Reagent added. The reaction was carried out for 30 min at RT, and the absorbance at 490 nm was measured using a microplate reader (Infinite^®^ 200 PRO, TECAN) after stopping the reaction. Data were reported as fold increase with respect to vehicle-treated cells (0.05% DMSO).

### 4.5. NRU Assay

The effect of AM on neutral red uptake (NRU) into lysosomes of viable cells was evaluated in cells cultured in 96-well plates and treated for 24 h with increasing concentrations of the compound. NRU was determined according to Borenfreund and Puerner (1985) [47], with some modifications. Following exposure to drug, the medium was removed, and neutral red dye (100 μg/mL, Sigma-Aldrich) was added to each well. After incubation at 37 °C for 90 min, cells were washed with PBS, and dye extraction solution (Ethanol/Water/Acetic Acid 50:49:1) was added. After 20 min of gentle shaking, the absorbance at 550 nm was measured using a microplate reader (Infinite^®^ 200 PRO, TECAN). Data were reported as percentage with respect to vehicle-treated cells (0.05% DMSO).

### 4.6. Phospholipidosis Evaluation Using NBD-PC Uptake

The phospholipidosis induced by AM treatment was assessed by the accumulation of a synthetic fluorescent phospholipid analogue, 1-acyl-2-[12-(7-nitro-2,1,3-benzoxadiazol-4-yl)amino]dodecanoyl]-glycero-3-phosphocholine (NBD-PC, Avanti Polar Lipids, Inc., Alabaster, AL, USA), according to Kasahara et al. [48]. In brief, cells were cultured in 96-well black clear bottom plates and treated for 24 h with AM in the presence of 40 μM NBD-PC. After 24 h, cells were washed twice with PBS and NBD-PC fluorescence measured (excitation and emission wavelengths of 485 and 538 nm, respectively) on an Infinite^®^ 200 PRO microplate reader (TECAN). Cells were then incubated for 20 min at 37 °C with Hoechst 33,342 solution (20 μg/mL in PBS) before measuring Hoechst fluorescence (excitation and emission wavelengths of 355 and 460 nm, respectively). Values for NBD-PC fluorescence were normalized to those of Hoechst fluorescence. Data were reported as folds increased with respect to vehicle-treated cells (0.05% DMSO).

### 4.7. Lysosomal β-Hexosaminidase A Assay

The effect of AM on the secretion of the lysosomal β-hexosaminidase A (HexA) was evaluated by measuring its specific enzymatic activity in the cell media. Cells were cultured in 6-well plates and treated for 24 h with increasing concentrations of the compounds. Cell culture medium (about 1.5 mL) was collected from each well and centrifuged at maximum speed in a bench centrifuge at 4 °C to eliminate cell debris. Supernatant (20 μL) was transferred in a 96-well black plate and mixed with 40 μL of 3 mM solutions of the synthetic fluorogenic substrate 4-methylumbelliferyl-β-N-acetyl-glucosaminide-6-sulfate (MUGS, Toronto Research Chemicals, Toronto, Canada) [49]. Assays were incubated for 1 h at 37 °C and then stopped with 290 μL of 0.4 M glycine-NaOH (pH 10.4). Fluorescence of the liberated 4-methylumbelliferone (excitation and emission wavelengths of 360 and 450 nm, respectively) was measured with an Infinite F200 fluorimeter (TECAN). One enzymatic unit is the amount of enzyme that hydrolyzes 1 mmol of substrate/min at 37 °C. Culture medium enzymatic activity was expressed as mU/mL media. Data were reported as fold increase with respect to vehicle-treated cells (0.05% DMSO). To evaluate whether HexA was released upon AM treatment as soluble or EV-associated protein, enzymatic activity measurement was carried out on the 10K pellet, 100K pellet and 100K supernatant. In brief, EVs were isolated as reported above from the same number of 8 μM AM-treated and vehicle-treated cells (0.05% DMSO), and then 20 μL of 10K pellet, 100K pellet or 100K supernatant were transferred in a 96-well black plate and mixed with 40 µL of MUGS solutions. Assays were incubated as above, and the fluorescence measured with an Infinite F200 fluorimeter.

### 4.8. Fluorescence Microscopy

Cells were seeded onto glass cover slips and grown for 24 h, washed three times with PBS, and then fixed with 4% paraformaldehyde/PBS for 15 min at RT. Cover slips were rinsed three times with PBS and mounted on glass slides using Vectashield with DAPI (Vector Laboratories, Burlingame, CA, USA). Fluorescence microscopy images were acquired with a Nikon TE2000 microscope (Nikon, Tokyo, Japan) through a 20× objective.

### 4.9. Immunoblotting

Cells were lysed at 4 °C in RIPA buffer (50 mM Tris-HCl pH 8, 150 mM NaCl, 1% (*v*/*v*) NP-40, 0.1% (*w*/*v*) SDS, 0.5% (*w*/*v*) sodium deoxycholate) in the presence of protease inhibitor cocktail (Merck Life Sciences, Darmstadt, Germany). Insoluble material was removed by centrifugation at 13.000× *g* for 10 min at 4 °C. Cell lysates or EV samples (~5–30 μg or 3–10 μg, respectively) were mixed with 5× loading buffer (1 M Tris-HCl pH 6.8, 5% (*w*/*v*) SDS, 6% (*v*/*v*) glycerol, 0.01% (*v*/*v*) Bromophenol blue) containing 125 mM DTT. Samples were boiled for 5 min, electrophoresed on acrylamide gel, and transferred to PVDF membrane at 100 V for 1 h. Rabbit polyclonal anti-mCherry (AB167453), rabbit polyclonal anti-GFP (AB1218), goat polyclonal anti-TFEB and mouse monoclonal anti-GAPDH were from Abcam (Cambridge, UK); mouse monoclonal anti-Alix antibody and goat polyclonal anticalnexin antibody were from Santa Cruz Biotechnology (Santa Cruz, CA, USA); mouse monoclonal anti-β-actin was from Sigma-Aldrich (St Louis, MI, USA); rabbit polyclonal anti-CD71 was from Biorbyt (Cambridge, UK) or Thermofisher scientific (Waltham, MA, USA); rabbit polyclonal anti-p62 and rabbit polyclonal anti-LC3B were from Cell Signaling Technology (Danvers, MA, USA); and mouse monoclonal NBR1 was from Abnova (Taipei, Taiwan, China). Mouse antigoat (Sigma-Aldrich), donkey antigoat (Santa Cruz), goat antirabbit and horse antimouse HRP-linked secondary antibodies (Cell Signaling) were probed according to manufacturer’s instructions. Immunoblots were detected by chemiluminescence using ECL system (Life Technologies, Carlsbad, CA, United States). Chemiluminescent signals were either captured on film or detected with a CCD camera (ChemiDoc XRS, Bio-Rad). Densitometric evaluation was obtained with ImageJ software.

### 4.10. Cells Preparation for Phospholipid Profile

For lipidomic analysis, cells were treated with AM at the indicated concentration for 24 h, and then they were trypsinized, washed twice with PBS at 4 °C, and centrifuged again. Approximately 3 × 10^6^ of either treated cells or untreated cells as control were pelleted and stored at −80 °C prior to analysis. Total cellular lipids were extracted from 3 different cell pellets and protein concentration determined in each sample to normalize lipid content. In the case of EVs, they were obtained from cell culture medium of the same three 3 different preparations used for cell lipid extraction. Lipid extraction was carried out as previously reported [50]. Extracts were dried under nitrogen and resuspended in a mixture 9:1 of methanol/toluene prior to be submitted for analyses.

### 4.11. Phospholipid Profile by Liquid Chromatography-Tandem Mass Spectrometry (LC-MS)

Phospholipid profile was conducted according to the method described by Tsugawa, H et al. [51] with minor modification. The LC system consisted of an Agilent 1260 Infinity II (Agilent Technologies, Santa Clara, CA, USA) with a pump, a column oven and an autosampler. Lipids were separated on an Agilent InfinityLab Poroshell120 EC-C18 column (100 × 3.0 mm; 2.7 μm). Column was maintained at 50 °C at a flow-rate of 0.6 mL/min. The mobile phases consisted of (A) 9:1 (*v*/*v*) water:methanol with 10 mM ammonium acetate and 0.2 mM ammonium fluoride and (B) 2:3:5 (*v*/*v*/*v*) acetonitrile:methanol/isopropanol with 10 mM ammonium formate and 0.2 mM ammonium fluoride. A sample volume of 5 μL was used for the injections in ESI(+) and ESI(−). Separation was conducted under the following gradient: 0 min 70% (B); 1 min 70% (B); 3.5 min 86% (B); 10.0 min 86% (B); 11.0 min 100% (B); 17.0 min 100% (B); 17.1 min 70% (B); and 19.0 min 70% (B). Mass spectrometric detection of lipids was performed on a 6530 Q-TOF (Agilent Technologies, Santa Clara, CA, USA) using an Iterative Data Dependent Acquisition method. The instrument was tuned using a reference mass m/z 121.050873, m/z 922.009798 (+) and m/z119.03632, m/z 980.016375 (−). Raw data were processed by MS-DIAL 4 software [51]. Lipid species annotation and quantification were conducted according to guidelines of the Lipidomics Standard Initiative (LSI) [https://lipidomics-standards-initiative.org/ accessed on December 2020] using SPLASH lipidomics I (Avanti Polar Lipids) as internal standards. Quantitative data were normalized on the protein content of cells or vesicles.

## Data Availability

Not applicable.

## Supplementary Materials

The following are available online at https://www.mdpi.com/article/10.3390/ijms222312922/s1.
