# Supplementary material for: Drug-Induced Lysosomal Impairment Is Associated with the Release of Extracellular Vesicles Carrying Autophagy Markers
Source: Int J Mol Sci. 2021 Nov 29;22(23):12922. doi: 10.3390/ijms222312922 (PMC8657686; doi:10.3390/ijms222312922)
Supplement: Supplementary file 1 [file ijms-22-12922-s001.zip › ijms-1478794-supplementary.pdf]

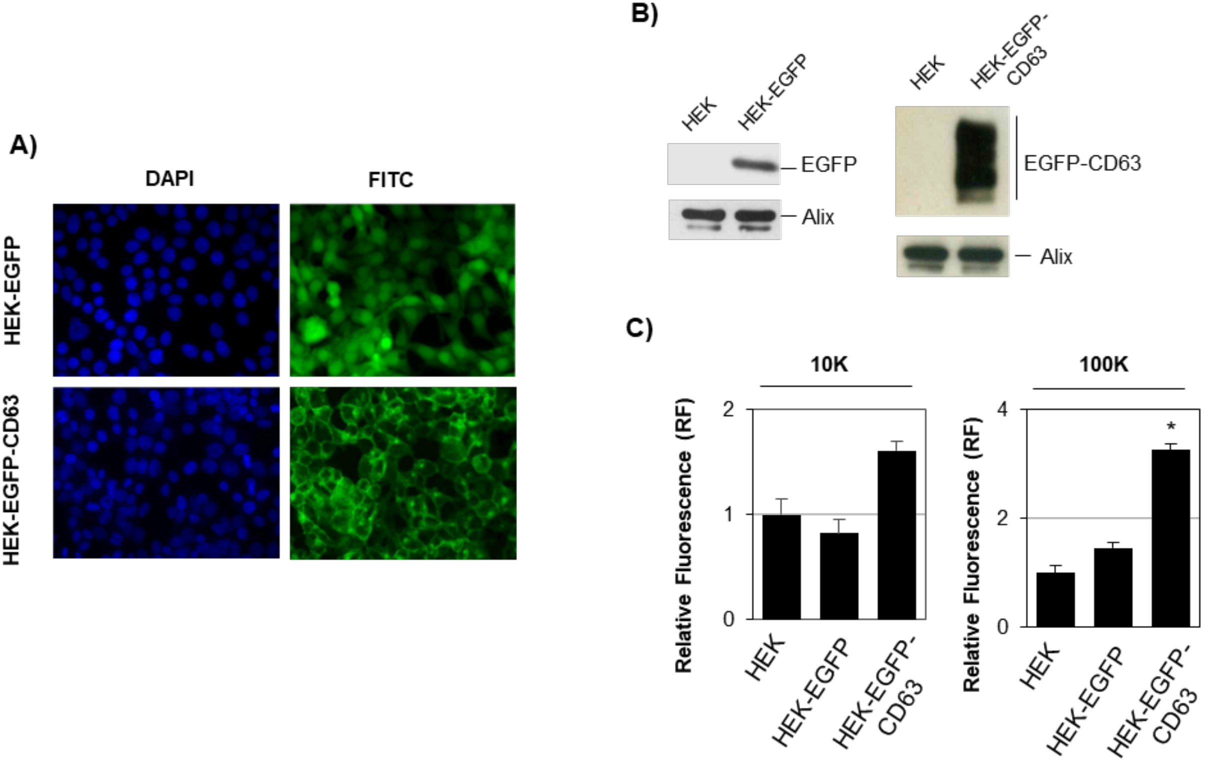

Supplementary Figure S1. HEK cells expressing EGFP release fluorescent EVs. A) Fluorescence microscopy. HEK cells were either transfected with EGFP or with CD63-EGFP and selected with blasticidin-S. Cells were grown onto glass coverslips, fixed with 4% paraformaldehyde and stained for nuclei with DAPI. Magnification 20X. B) Fluorescence analysis. Cell culture medium was centrifuged at 2, 000 g for 10 min and 100 µl were assayed for fluorescence at 485 nm ex, 520 em. C) Immunoblotting analysis of EGFP in cells. Extracts were sized by SDS-PAGE and probed with an anti-GFP antibody. As internal control, the membrane was also probed with an anti-Alix antibody. The densitometric analysis of results is reported below. Bars represent the ratio between the intensity of the EGFP and Alix signals. They are expressed as Relative intensity. D) Fluorescence analysis of EVs. EVs obtained by centrifugation at 10,000 g (10K) and at 100,000 g (100K). Pellets were resuspended in 100 µl PBS and assayed for fluorescence at 485 nm ex, 520 em. *P<0.01 with respect to untransfected HEK cells as control.
